# The correlation of radiographic findings and patient symptomatology in cervical degenerative joint disease: a cross-sectional study

**DOI:** 10.1186/s12998-015-0052-0

**Published:** 2015-02-09

**Authors:** Iris Sun Rudy, Alexandra Poulos, Laura Owen, Ashlee Batters, Kasia Kieliszek, Jessica Willox, Hazel Jenkins

**Affiliations:** Macquarie University, Sydney, Australia

**Keywords:** Degenerative joint disease, Osteoarthritis, Cervical spine, Neck pain, Neck stiffness, Sensitivity and specificity

## Abstract

**Background:**

There are few known studies investigating the correlation of symptomatology with the specific subtypes of cervical spine degenerative joint disease demonstrated on radiograph. The aim of this study was to assess the correlation and diagnostic test accuracy of specific symptoms in determining the presence, type and severity of degenerative joint disease on radiograph.

**Methods:**

A retrospective cross-sectional design was used to correlate cervical radiographic findings with neck pain and related symptomatology. Radiographs of 322 patients from April 2010 to June 2012 were assessed and evidence of radiographic cervical degenerative joint disease was extracted. Clinical data for each patient was obtained from their patient files including: pain using a VAS, presence of neck stiffness, presence of headaches, presence of shoulder referral, presence of hand radiculopathy and presence of hand numbness. Measures of diagnostic test accuracy and regression analysis were used to assess for any correlation between symptoms and radiographic findings.

**Results:**

Referral of pain to the shoulder and neck stiffness showed small degrees of correlation with cervical degenerative joint disease, however, these correlations were not maintained when age was accounted for. Only age showed consistent statistical significance as a predictor for degree of disc degeneration (correlation coefficient (95% confidence interval): 0.06 (0.055, 0.066)); the presence of facet hypertrophy (odds ratio (95% confidence interval): 1.12 (1.09, 1.15)); or uncinate process hypertrophy (odds ratio (95% confidence interval): 1.15 (1.12, 1.18)). Neck stiffness demonstrated a small degree of diagnostic test accuracy for the degree of cervical disc degeneration (area under the curve (95%CI): 0.62 (0.56, 0.68)) and the presence of either facet (diagnostic OR (95%CI):1.69 (1.04, 2.76)) and uncinated process hypertrophy (LR+ (95%CI): 1.17 (1.00, 1.38)).

**Conclusion:**

The results of this study indicate that clinical symptoms such as pain level, headaches, shoulder referral and hand radiculopathy or numbness are not reliably correlated with radiographic findings of degenerative joint disease in the cervical spine. A small increase in diagnostic accuracy between the presence of neck stiffness and all forms of cervical degenerative joint disease is shown, however, this increase is not at the level expected to change clinical practice.

## Background

Neck pain is a widespread entity that affects a large majority of the population. The prevalence is greatest amongst middle-aged people, with approximately 66% of individuals experiencing neck pain and related symptoms at some stage in their lives [1,2]. Neck pain is also the second most frequent musculoskeletal complaint presenting to primary healthcare practitioners [3] and most people with neck pain do not experience a complete resolution of symptoms [4]. Although there is a large prevalence of neck pain, neck pain is difficult to diagnose [4] and therefore, to treat.

Among the many causes of neck pain, degenerative joint disease of the cervical spine is a common condition affecting the synovial joints [5] with a prevalence of 3.3 cases per 1000 people [6]. It is characterised by a series of degenerative changes comprising intradiscal tears with subsequent disc space loss, osteophytic growths and spur formation, ligamentous hypertrophy and capsular thickening [7]. Cervical spine degenerative joint disease occurs mostly in 4^th^ and 5^th^ decades and is associated with the natural aging process [8]. It also varies in presentation, with the most common complaints consisting of neck pain, activity related neck stiffness, headaches and upper limb referral [7].

Magnetic resonance imaging (MRI) remains the most advanced assessment tool to evaluate degenerative changes, however the costs and accessibility to this type of imaging requires clinicians to differentiate those patients that require this level of investigation. Plain film radiography is the most commonly used modality to diagnose degenerative joint disease [9], however the literature indicates that radiographic findings do not correlate well with pain and symptoms [10,11]. It also has been suggested that the use of plain film radiography based solely on the suspicion of detecting degenerative changes is not clinically justified [12,11].

Marchiori and Henderson [13] compared radiographic findings of spinal degeneration with severity and chronicity of cervical pain and any resulting lifestyle changes in 700 consecutive patients referred for a cervical radiographic examination. The authors concluded that increasing levels of spinal degeneration are related to increased chronicity of patient complaints. Gore et al. [14] in 1987 completed an investigation in 205 patients previously seen for a neck complaint. A follow up radiographic examination was conducted after a minimum of ten years to assess for subsequent spinal degeneration. The results concluded no significant relationship between the degree of spinal degeneration and patient symptoms at either initial presentation or at follow up. Heller et al. [11] examined the relation between symptoms and changes seen on radiograph in a hospital setting. Using the symptoms of pain in the arm or shoulder, shoulder blade, neck, and back of the head and stiffness in the neck, they concluded that there were few and inconsistent relationships between symptoms and changes on radiograph. However, they did not examine whether hallmark symptoms could be predicting factors in diagnosing the severity or grade of cervical degenerative joint disease.

The aim of this study was to assess for correlation and the diagnostic test accuracy of hallmark symptoms in determining the presence, type and severity of cervical degenerative joint disease on radiograph.

## Methods

### Sample selection

The subjects included in this study were patients attending one of three Macquarie University Chiropractic Teaching Clinics who had cervical radiographs taken between April 2010 to June 2012. Of the 502 studies taken in this period, 180 were excluded, as they were: under 18 years of age, had incomplete or absent patient files or the cervical radiographic quality was too poor to interpret (non-diagnostic). This study complies with national research ethics guidelines and was approved by the Macquarie University Human Research Ethics Committee (HREC), approval number: 5201300294.

### Data collection

Two members of the research team accessed the chiropractic clinic’s radiographic report database to find all cervical spine radiographs taken within the selected time period. The radiographic reports were assessed and the following information was recorded: name, date of birth, gender and presence of degenerative joint disease (Yes/No) on cervical anteroposterior (AP) and lateral views. The AP and lateral radiographs reported to have signs of cervical degenerative joint disease were then accessed and graded using the Kellgren and Lawrence Osteoarthritis Severity Grading System [15-17] by the same two research members. The presence of facet hypertrophy and uncinate process hypertrophy was also recorded (Yes/No). The assessed radiographic reports were reported by chiropractic radiologists at the time of imaging. The two members of the research team assessing the radiographs for the grading of degenerative joint disease were both in their final year of chiropractic studies.

The names of the included patients were then provided to three other research team members, also in their final year of chiropractic studies, uninvolved with the radiographic data collection to preserve blinding. These researchers located the corresponding patient files in the clinics and extracted clinical data taken at the time of cervical radiographic referral, relating to the following: cervical pain levels (measured on the visual analogue scale (VAS)); the presence (Yes/No) as reported by the patient of: headaches, neck stiffness, pain referral to shoulders, radicular symptoms to the hands and numbness in the hands. Data pertaining to initial consultation, previous treatment or management plans was not recorded.

The recorded data was allocated to an independent group member, also in their final year of chiropractic studies, with no involvement in the radiographic or clinical data collection processes, to analyse and compare the results. Intra- and inter-observer reproducibility on the radiographic evaluations and extraction of clinical data was not performed, however, the radiographic evaluations were performed independently by two members of the research team. Results were compared and any discrepancies were discussed and arbitrated by the group supervisor, a chiropractic radiologist. Extraction of clinical data was from standardised files used in a chiropractic teaching institution. Files were excluded from analysis if they had incomplete or ambiguous data with respect to the symptoms being assessed.

### Instruments

#### Kellgren and Lawrence osteoarthritis severity grade

Disc degeneration is a component of cervical degenerative joint disease. Kellgren developed certain criteria to classify and grade disc degeneration in the spine [15-17]. It uses the presence and severity of osteophytes, disc space narrowing and sclerosis to grade the level of degenerative disc disease present within a vertebral level on a grade of 1to 4 as outlined in Table 1. This assessment tool was selected based on its accuracy, reproducibility and recent validation for osteoarthritis in the cervical spine [18].

**Table 1:** **Kellgren-Lawrence grading scale** [15]

Grade 0	No signs of degenerative disc disease
Grade 1	Minimal anterior osteophytes
Grade 2	Definite anterior osteophytosis with possible narrowing of the disc space and some sclerosis of vertebral plates
Grade 3	Moderate narrowing of the disc space with definite sclerosis of vertebral plates and osteophytes
Grade 4	Severe narrowing of the disc space with definite sclerosis of vertebral plates and multiple large osteophytes

#### Presence of facet joint hypertrophy and uncinate process hypertrophy

These radiographic findings are also components of cervical degenerative joint disease [5]. Disc degeneration, facet joint hypertrophy and uncinate process hypertrophy may occur independently or in combination with each other. There is no known grading scale for facet joint and uncinate process hypertrophy, therefore the presence or absence of these findings was assessed (Yes/No).

#### Visual Analogue Scale (VAS)

The VAS is a subjective measurement of a person’s pain intensity on a scale of 0 (no pain) to 10 (worst pain you have ever felt). This scale was used as it’s a commonly implemented tool for patients presenting at the student clinics. The VAS is also considered valid, reliable and appropriate for clinical use [19,20].

#### Presence of headaches, neck stiffness, referral to shoulder, radicular symptoms in hands and numbness in hands

These symptoms are common complaints associated with cervical degenerative joint disease which are well described in the literature [5]. The presence of these symptoms was recorded (Yes/No) as reported in the clinical files at the time of radiographic referral.

### Data analysis

Linear or logistic regression analysis was used to assess for correlations between radiographic findings of cervical degenerative joint disease as the dependent variable and independent predictor variables. The independent predictor variables assessed were: gender, age, VAS, presence of neck stiffness, presence of headache, presence of shoulder referral and presence of hand radiculopathy or numbness.

Diagnostic accuracy testing was used to assess dichotomous data. Dichotomous data included the presence of facet hypertrophy and uncinate hypertrophy as dependent variables. These were tested for positive and negative correlations to the presence or absence of headache, neck stiffness, shoulder referral, hand radiculopathy and hand numbness as predictor variables. Sensitivity, specificity, positive predictive value (PPV), negative predictive value (NPV), positive likelihood ratio (LR+), negative likelihood ratio (LR-) and diagnostic odds ratios (OR) were calculated with 95% confidence intervals (95%CI).

ROC curves were used to assess for the diagnostic test accuracy of continuous data compared to dichotomous data. Comparisons were made between the grade of disc degeneration as a dependent variable and the presence of headache, shoulder pain, neck stiffness, hand radiculopathy or hand numbness as independent predictor variables. The area under the ROC curve with 95%CI was calculated to assess for statistical significance of the diagnostic test accuracy.

## Results

322 subjects were included in the study. Of these, 162 (50.3%) subjects were male, the mean age was 40.5 (standard deviation 17.4) and 78 (24.2%) subjects had no radiographic signs of cervical degenerative joint disease. Table 2 outlines the distribution of radiographic findings degenerative joint disease in the cervical spine.

**Table 2:** **Distribution of radiographic findings of cervical spine degenerative joint disease**

**Disc degeneration**		**Facet hypertrophy**		**Uncinate process hypertrophy**	
**Kellgren Lawrence grade**	**No. (%)**	**Presence (Y/N)**	**No. (%)**	**Presence (Y/N)**	**No. (%)**
0	78 (24.2)	Y	118 (36.6)	Y	141 (43.8)
1	101 (31.4)	N	204 (63.4)	N	181 (56.2)
2	54 (16.8)				
3	45 (14)				
4	44 (13.7)				
Total	322 (100)	Total	322 (100)	Total	322 (100)

No.: Number of subjects exhibiting the finding; Y: Yes; N: No.

### Kellgren Lawrence grade of cervical disc degeneration

**Figure 1:**
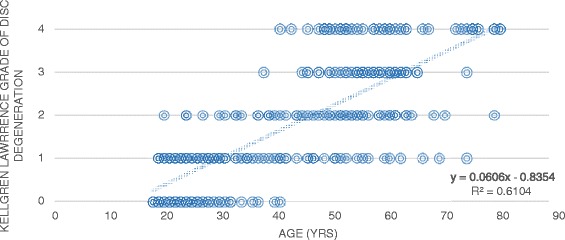
**Correlation of disc degeneration vs age.**

Linear regression analysis was conducted to examine the relationship between the Kellgren Lawrence grade of disc degeneration and the independent predictor variables. A significant trend could only be extrapolated for age as an independent variable. As demonstrated in Figure 1, the correlation coefficient (95%CI) of 0.06 (0.055, 0.066) shows a positive trend and variation around the regression line is acceptable as indicated by the R^2^-value of 0.61. This indicates that for every 16.7 years a person ages, their grade of cervical disc degeneration is expected to increase by 1 on the Kellgren Lawrence grading scale.

**Figure 2:**
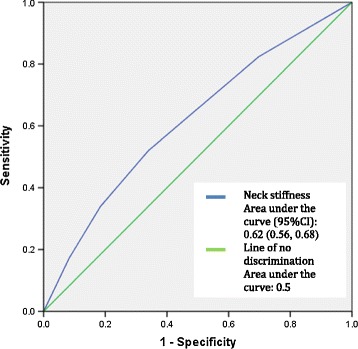
**ROC curve for presence of neck stiffness vs grade of disc degeneration.**

**Figure 3:**
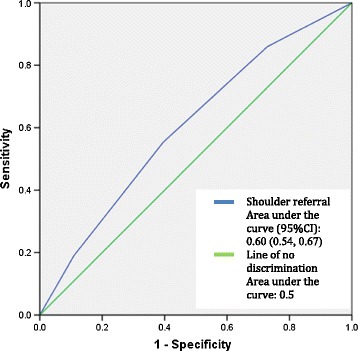
**ROC curve for presence of shoulder referral vs grade of disc degeneration.**

Assessment of diagnostic accuracy between the Kellgren Lawrence grade of disc degeneration and specified symptomatologies was performed using ROC curves. The presence of neck stiffness and the presence of shoulder referral did show mild evidence of diagnostic accuracy in predicting disc degeneration as depicted in Figures 2 and 3. The area under the curve (95%CI) measured 0.62 (0.56, 0.68) and 0.60 (0.54, 0.67) respectively. Although these values were statistically significant the clinical significance is uncertain as these values do not represent high levels of diagnostic accuracy. The area under the curve (Area under curve (95%CI)) for headaches (0.43 (0.36, 0.50)), hand radiculopathy (0.57 (0.47, 0.67)) and hand numbness (0.57 (0.47, 0.68)) did not exhibit statistical significance. Therefore, the presence of headaches, hand radiculopathy and hand numbness did not predict the grade of disc degeneration on cervical radiograph.

### Presence of cervical facet joint hypertrophy

Logistic regression analysis was conducted to examine the relationship between presence of facet joint hypertrophy and the independent predictor variables. Only age showed a significant relationship with an odds ratio (95%CI) of 1.12 (1.09, 1.15). Therefore, for every year increase in age the odds of having facet hypertrophy increase by 1.12.

Table 3 depicts the diagnostic accuracy outcomes for the presence of facet joint hypertrophy and the presence of specified symptomatologies. The presence of neck stiffness was the only symptom to demonstrate statistically significant diagnostic accuracy with diagnostic OR (95%CI) of 1.69 (1.04, 2.76). Despite being statistically significant, however, this only represents a small increase in the odds of having facet hypertrophy on radiograph in the presence of neck stiffness. Hand radiculopathy and hand numbness both exhibited a high specificity for the presence of facet joint hypertrophy on radiograph at 0.89 (0.84, 0.93) and 0.90 (0.85, 0.94) respectively. Therefore, if there is no facet joint hypertrophy on radiograph then it is unlikely that the patient will have presented with hand radiculopathy or hand numbness.

**Table 3:** **Diagnostic accuracy of specified symptomatologies for facet joint hypertrophy**

	**Sensitivity (95%CI)**	**Specificity (95%CI)**	**PPV (95%CI)**	**NPV (95%CI)**	**LR+ (95%CI)**	**LR- (95%CI)**
Headache	0.28 (0.21, 0.37)	0.65 (0.59, 0.71)	0.32 (0.24, 0.42)	0.61 (0.54, 0.67)	0.81 (0.57, 1.14)	1.1 (0.95, 1.28)
Neck stiffness	0.71 (0.63, 0.79)	0.4 (0.34, 0.47)	0.41 (0.34, 0.48)	0.71 (0.62, 0.78)	1.2 (1.02, 1.41)	0.71 (0.51, 0.98)
Shoulder referral	0.41 (0.32, 0.5)	0.64 (0.57, 0.7)	0.4 (0.31, 0.49)	0.64 (0.57, 0.71)	1.13 (0.85, 1.5)	0.93 (0.77, 1.11)
Hand Radiculopathy	0.14 (0.09, 0.21)	0.89 (0.84, 0.93)	0.42 (0.28, 0.58)	0.64 (0.58, 0.69)	1.26 (0.69, 2.3)	0.99 (0.89, 1.06)
Hand Numbness	0.1 (0.06, 0.17)	0.9 (0.85, 0.94)	0.36 (0.22, 0.53)	0.63 (0.58, 0.69)	0.99 (0.5, 1.94)	1.0 (0.93, 1.08)

### Presence of cervical uncinate process hypertrophy

Logistic regression analysis was conducted to examine the relationship between presence of uncinate process hypertrophy and the independent predictor variables. Only age showed a significant relationship with an odds ratio (95%CI) of 1.15 (1.12, 1.18). Therefore, for every year increase in age the odds of having uncinate process hypertrophy increase by 1.15.

Table 4 depicts the diagnostic accuracy outcomes for the presence of uncinate process hypertrophy and the presence of specified symptomatologies. Neck stiffness demonstrated a small positive correlation with the presence of uncinate process hypertrophy with a sensitivity (95% CI) of 0.70 (0.61, 0.77) and LR+ (95% CI) of 1.17 (1.00, 1.38). Therefore, the presence of neck stiffness correlates with a mild increase in the odds of having uncinate process hypertrophy on radiograph. Hand radiculopathy and hand numbness both exhibited a high specificity for the presence of uncinate process hypertrophy on radiograph at 0.91 (0.85, 0.94) and 0.92 (0.87, 0.95) respectively. Therefore, if there is no uncinate process hypertrophy on radiograph then it is unlikely that the patient will have presented with hand radiculopathy or hand numbness.

**Table 4:** **Diagnostic accuracy of specified symptomatologies for uncinate process hypertrophy**

	**Sensitivity (95% CI)**	**Specificity (95% CI)**	**PPV (95% CI)**	**NPV (95% CI)**	**LR+ (95% CI)**	**LR- (95% CI)**
Headache	0.3 (0.24, 0.39)	0.66 (0.59, 0.73)	0.42 (0.33, 0.51)	0.55 (0.48, 0.61)	0.91 (0.66, 1.26)	1.05 (0.9, 1.22)
Neck stiffness	0.7 (0.61, 0.77)	0.41 (0.34, 0.48)	0.48 (0.41, 0.55)	0.63 (0.54, 0.71)	1.17 (1.0, 1.38)	0.75 (0.55, 1.01)
Shoulder referral	0.44 (0.36, 0.52)	0.66 (0.59, 0.73)	0.5 (0.42, 0.59)	0.6 (0.53, 0.66)	1.23 (0.98, 1.7)	0.85 (0.71, 1.02)
Hand Radiculopathy	0.15 (0.1, 0.22)	0.91 (0.85, 0.94)	0.55 (0.4, 0.7)	0.58 (0.52, 0.63)	1.59 (0.87, 2.89)	0.94 (0.86, 1.02)
Hand Numbness	0.13 (0.08, 0.19)	0.92 (0.87, 0.95)	0.55 (0.38, 0.7)	0.57 (0.52, 0.63)	1.54 (0.81, 2.95)	0.95 (0.88, 1.03)

## Discussion

In this study, symptomatology was not correlated with the presence of cervical degenerative joint disease. The only independent predictor to show consistent significant correlation with radiographic findings of degenerative joint disease was age. Cervical pain levels, measured by VAS, and the presence of headaches did not provide any increase in diagnostic accuracy for the presence of cervical degenerative joint disease. Hand radiculopathy and hand numbness did not show any correlations with grade of degenerative disc disease, however they did exhibit high specificity for the presence of facet joint or uncinate process hypertrophy. Although a correlation between radiographic findings and symptomatology is evident here, clinically this result does not aid the clinician in predicting the presence or absence of cervical degenerative joint disease from the presence or absence of these symptoms.

The presence of pain referring to the shoulder exhibited statistically significant but low diagnostic test accuracy with the grade of degenerative disc disease in the cervical spine. The area under the ROC curve, despite being statistically significant at 0.60 (95%CI: 0.54, 0.67), only represents a low level of accuracy in predicting radiographic findings of degenerative disc disease from the presence of shoulder referral. Similarly the accuracy of the presence of neck stiffness in predicting cervical disc degeneration also had an area under the ROC curve of 0.62 (95%CI: 0.55, 0.68), indicating a statistically significant result but of low diagnostic accuracy.

As a symptom, the presence of neck stiffness showed mild diagnostic test accuracy with all radiographic findings associated with degenerative joint disease of the cervical spine. The correlation of the presence of neck stiffness with radiographic evidence of cervical degenerative joint disease is plausible considering the combination of hypertrophic changes to the articular surfaces of the joints, osteophyte formation and resulting muscle tension that can cause neck stiffness [21]. However, the LR+ for both facet joint and uncinate process hypertrophy, despite exhibiting statistical significance, were at values associated with low levels of diagnostic accuracy that would not be expected to change clinical practice [22]. In addition, when regression analysis was performed, and age and gender were also accounted for, neck stiffness did not show statistically significant correlation with any form of cervical spine degenerative joint disease.

The results of this study agree with previous studies that the severity of cervical degeneration is not correlated with the degree of pain perceived [3,10,12,14]. This study adds to the body of evidence in this area by assessing the different radiographic findings associated with cervical degenerative joint disease individually and by grading the extent of degenerative disc disease using established grading criteria. Symptoms associated with cervical degenerative joint disease were also assessed individually for diagnostic accuracy. Although results were similar between disc degeneration, facet joint hypertrophy and uncinate process hypertrophy, some statistically significant differences were noted. The grade of disc degeneration correlated most significantly with the presence of neck stiffness or referral of pain to the shoulder, whereas, facet joint and uncinate process hypertrophy showed correlation with the presence of neck stiffness and high specificity with upper limb radicular symptoms.

The main limitation of this study is the retrospective study design. Only subjects who had been referred for cervical imaging were assessed and this would have skewed the sample towards subjects with symptomatology. In addition, clinical files were accessed to obtain data regarding presenting symptoms and, therefore, inter-practitioner inconsistencies when recording symptomatic data at the time of presentation could not be accounted for. Any files with missing data were excluded from the study to limit the associated bias. A prospective study would control for these limitations, however, the results of this study do not provide compelling evidence that a prospective study in this field would produce results that would lead to a change in clinical practice. Finally, this study only assessed for the presence of degeneration in any one of cervical discs, facet joints or uncovertebral joints. It did not account for presentations of multi-level or concomitant degenerative findings. These additional findings may help differentiate between levels of severity of cervical degeneration, however, a validated scale for this assessment could not be found in the current literature. Validation of a more precise scale to grade the severity of cervical degeneration may allow for further research into this area and a better understanding of any association between symptomatology and cervical degenerative joint disease.

## Conclusion

In conclusion, the results of this study indicate that clinical symptoms such as pain level (VAS), headaches, shoulder referral and hand radiculopathy or numbness do not correlate with radiographic findings of degenerative joint disease in the cervical spine. A small increase in diagnostic accuracy between the presence of neck stiffness and all forms of cervical degenerative joint disease was shown. However, this increase is not at the level expected to change clinical practice. Age was the only independent predictor variable to demonstrate a statistically significant correlation with radiographic findings of cervical spine degenerative joint disease.

### Consent

Written informed consent was obtained from the patient for the publication of this report and any accompanying images.

## Abbreviations

AP: Anteroposterior
VAS: Visual analogue scale
PPV: Positive predictive value
NPV: Negative predictive value
LR+: Positive likelihood ratio
LR-: Negative likelihood ratio
OR: Odds ratio
95%CI: 95% confidence intervals
